# Fast Thermal Calibration of Low-Grade Inertial Sensors and Inertial Measurement Units

**DOI:** 10.3390/s130912192

**Published:** 2013-09-12

**Authors:** Xiaoji Niu, You Li, Hongping Zhang, Qingjiang Wang, Yalong Ban

**Affiliations:** GNSS Research Center, Wuhan University, No.129, Luoyu Rd., Wuhan 430079, Hubei, China; E-Mails: xjniu@whu.edu.cn (X.N.); liyou@whu.edu.cn (Y.L.); qjwangwhu@whu.edu.cn (Q.W.); ylban@whu.edu.cn (Y.B.)

**Keywords:** MEMS inertial sensors, IMU, thermal calibration, turntable, temperature chamber

## Abstract

The errors of low-cost inertial sensors, especially Micro-Electro Mechanical Systems (MEMS) ones, are highly dependent on environmental conditions such as the temperature. Thus, there is a need for the development of accurate and reliable thermal compensation models to reduce the impact of such thermal drift of the sensors. Since the conventional thermal calibration methods are typically time-consuming and costly, an efficient thermal calibration method to investigate the thermal drift of a full set of gyroscope and accelerometer errors (*i.e.*, biases, scale factor errors and non-orthogonalities) over the entire temperature range in a few hours is proposed. The proposed method uses the idea of the Ramp method, which removes the time-consuming process of stabilizing the sensor temperature, and addresses its inherent problems with several improvements. We change the temperature linearly for a complete cycle and take a balanced strategy by making comprehensive use of the sensor measurements during both heating and cooling processes. Besides, an efficient 8-step rotate-and-static scheme is designed to further improve the calibration accuracy and efficiency. Real calibration tests showed that the proposed method is suitable for low-grade IMUs and for both lab and factory calibration due to its efficiency and sufficient accuracy.

## 1. Introduction

Integrated Global Navigation Satellite System (GNSS)/Inertial Navigation Systems (INS) systems are becoming widely used techniques to provide precise and reliable navigation information (*i.e.*, position, velocity, attitude, *etc.*) in fields such as surveying and mapping, spatial information systems and intelligent transport [1]. A GNSS receiver is mainly used to provide high absolute position accuracy with long-term stability in ideal conditions, but has certain limitations in challenging areas (e.g., city downtowns, inside tunnels, under heavy tree canopies, *etc.*). On the contrary, as fully self-contained systems, INS measures the specific forces and angular rates by inertial sensors [*i.e.*, accelerometers and gyroscopes (gyros)] and determines the motion of a body with respect to an inertial frame of reference [2]. Due to the integration of the inertial measurement, the sensor errors will accumulate and grow, resulting in increasing position and velocity errors. Since GNSS and INS are complementary systems, they can be integrated to give a system that has several advantages over each individual component separately. In such integration, the GNSS-derived positions and velocities are updating information through a Kalman Filter (KF) while the IMU is used to provide the navigation information during GNSS signal outages and for fast GNSS signal reacquisition [3]. However, traditional INS devices are bulky, expensive and complex, which often precludes their use outside specialized application fields, especially in civilian areas [4].

During these decades, advances in Micro-Electro-Mechanical Systems (MEMS) technology combined with the miniaturization of electronics have made it possible to produce chip-based inertial sensors. These MEMS chips have become ideal candidates for various applications since they are small, light-weight, low power and are extremely low-cost and reliable [5]. At the same time, the cost reduction of GNSS receivers has also promoted the development of low-cost navigation techniques. Accordingly, low-cost GNSS receiver-chip/MEMS INS integrated systems are becoming widely-used navigation techniques.

However, the errors of the low-cost sensors, especially the MEMS ones, will change with time and are highly dependent on environmental conditions such as temperature [6]. Therefore, even though in-lab calibration at room temperature is known to be a useful way to remove the major part of the deterministic sensor errors, the actual values of the sensor errors can be different from those obtained through calibration processes due to the difference between the operational and calibration temperatures. In real tests, we find that the IMU sensor errors, not only biases, but also scale factors and non-orthogonalities, may vary significantly with temperature. These changes reach thousands of deg/h and many thousands of μg for gyros and accelerometers biases, and thousands of ppm for the sensor scale factors and non-orthogonalities. Such errors, if not compensated for, will accumulate and lead to attitude and position drifts [7]. Approximately, for 2-D navigation, uncompensated gyros and accelerometers biases result in position errors of (1/6)*b_g_* (*T*)*gt*^3^ and (1/2)*b_a_* (*T*)*t*^2^, respectively [5], where *b_g_*(*T*) and *b_a_*(*T*) are the gyro and accelerometer biases at temperature *T; t* is the time that INS work alone; *g* is the local gravity value. Also, the uncompensated sensor scale factor errors may introduce position errors proportional to time squared. Thus it is important to do thermal calibration for MEMS IMUs so as to develop accurate and reliable thermal models.

However, the conventional thermal calibration methods are typically time-consuming and costly. In this paper, an efficient thermal calibration method to investigate the thermal drift of a full set of gyroscope and accelerometer errors (*i.e.*, biases, scale factor errors and non-orthogonalities) over the entire temperature range in a few hours is proposed.

## 2. Previous Works

### 2.1. Calibration and Modeling of Sensor Errors

The inertial sensor errors can be divided into two types: deterministic (systematic) errors and stochastic errors. Calibration is an important way to remove the major part of the deterministic IMU sensor errors. Various IMU calibration methods have been proposed according to different grades of IMUs and different applications [2,8–12]. On the contrary, the stochastic errors can only be modeled instead of being calibrated and removed. Among various stochastic modeling methods, Allan Variance is commonly used to determine the characteristic of the underlying random processes [13].

The thermal drifts of the sensor errors are normally regarded as deterministic errors. To reduce the thermal drift of the sensor errors, the following two processes are needed: (1) Thermal calibration: to develop accurate and reliable thermal models of the sensor errors, *i.e.*, build the relationship between the sensor errors and the sensor temperature; (2) Thermal compensation: to compensate the thermal drift of the sensor errors according to their online temperature during the operation process of the IMUs. Both these processes are dependent on the temperature provided by the internal temperature sensors of the IMUs.

Official thermal calibration needs professional equipment such as a thermal chamber and a turntable (optional). Such professional equipment can be used to get reliable thermal calibration results over a large temperature range. However, when using these high-cost equipments, it is also important to improve the calibration efficiency, so as to reduce the equipment cost.

The purpose of thermal calibration is to determine the IMU sensor errors under different temperature points. Therefore, thermal calibration is commonly used based on the following assumption: the thermal drift of a sensor error is only related to the temperature of the sensor core. That is, corresponding to a certain temperature, the sensor errors will have the same values. Based on this assumption, the relationship between the sensor errors and the temperature can be built directly through either curve fit (as used in this paper) or a lookup table, if the corresponding sensor errors at different temperatures are known.

There are currently two main approaches for thermal calibration: the Soak method and the Ramp method [2]. The Soak method works on the premise of stable sensor temperature while the Ramp method works based on time-varying sensor temperature.

### 2.2. Temperature Soak Method

The Soak method calibration works as follows: (a) Stabilize the temperature of the sensors or IMUs at a certain temperature point; (b) Record the sensor measurements and calculate the sensor errors corresponding to such point; (c) Repeat this process at several typical temperature points, then a series of sensor errors along with temperature data can be obtained. After that, the IMU sensor errors at other temperature points can be calculated by interpolation [14]. The Soak method is used for both the calibration of deterministic errors and the modeling of stochastic errors [15–21]. By stabilizing the temperature, the Soak method can provide most reliable values of the sensor errors at the chosen temperature points. The main fact which restricts the use of the Soak method is the time required and the corresponding costs related to the equipment, time and manpower. The process of stabilizing the sensor temperature inside a MEMS IMU is usually time-costing.

### 2.3. Temperature Ramp Method

Different from the Soak method, the Ramp method calibration works as follows: (a) Control the temperature of the thermal chamber while it is continuously linearly increased or decreased to cover the full temperature range; (b) During this process the IMU measurements are recorded and the IMU sensor errors are calibrated [22,23]. The Ramp method is fundamentally faster in principle because it removes the stabilization process, and works based on linearly changed sensor temperature. However, there are two main inherent problems of the Ramp method, especially when it is used for the calibration of the IMUs, instead of the sensors:

*Issue #1*: It is not realistic to get the real temperature of the inertial sensors: there are temperature differences between the inertial sensors and the temperature sensors inside the IMUs because they have different cores. What we can get is the temperature at the core of the temperature sensor, not that of the inertial sensors. Moreover, such temperature differences will be different under various temperature changing conditions. This issue is similar to the hysteresis effect.
*Issue #2*: The chamber temperature changes during a calibration scheme (one set of IMU motions, with which the IMU outputs can be used to calculate for a set of IMU sensor errors; during the whole temperature calibration process, the turntable repeats the calibration scheme at different temperatures). This will lead to the changes of the IMU sensor errors to be calibrated, which in turn cause the calibration errors because the raw sensor data is not collected at the same temperature.

Both these two problems can impair the performance of the Ramp method. This can also explain why the process of stabilizing the temperature is needed in the Soak method. In this paper, we use the idea of the Ramp method, which removes the time-consuming process of stabilizing the temperature. Also, several ways to ease its inherent problems are considered. The details of the proposed method are shown in the next section.

## 3. Methodology

This section includes the basic idea of the proposed thermal calibration method, including the ways to deal with the inherent issues of the Ramp method, the calibration equipment used and the corresponding calibration scheme.

### 3.1. Basis Idea

We use the idea of the Ramp method, but consider the following ways to mitigate its inherent problems for improvement:

*Improvement #1*: take a balanced heating-and-cooling strategy to compensate the thermal calibration errors. To be specific, we change the temperature linearly for a complete cycle, and make comprehensive use of the sensor measurements under both the linear heating and cooling processes at the same changing rate to get the final thermal calibration results at the same temperature point.
*Improvement #2*: use the same calibration scheme during the whole calibration process. This can compensate the errors caused by the temperature changes during each calibration scheme to some extent after considering the heating and cooling processes comprehensively. In another word, the impact of the temperature changes in the calibration scheme during the heating process can be somehow cancelled with the impact during the cooling process at the same temperature point.
*Improvement #3*: narrow down the temperature change during each calibration scheme. One possible way for this is to calibrate the IMU sensor errors within the shortest period while the temperature is changing. We designed an efficient 8-step rotate-and-static scheme, which can calibrate the gyro and accelerometer biases, scale factors and non-orthogonalities of MEMS IMUs within only 2.5 min.

The corresponding explanations to these improvements can be stated as below:

*Explanation #1*: under the heating and cooling processes at the same temperature changing rate, the effects of the corresponding temperature differences between the inertial sensors and the temperature sensors can cancel each other approximately (*i.e.*, they have the similar value but opposite signs).
*Explanation #2*: using the same calibration scheme means that the impact of the temperature change at each sensor error can be cancelled to the maximum extent. For example, assume it is under heating process and the nominal temperature of the calibration scheme (*i.e.*, the average temperature during a scheme) is *T*_1_; when the IMU is rotated around the x-axis gyro clockwise, the temperature is *T*_1_ + d*T*_1_. Then, for the cooling process at the same temperature changing rate at *T*_1_, the IMU rotation around the x-axis gyro clockwise will happen at the temperature of *T*_1_ − d*T*_1_. Therefore the impacts of the temperature variations from the nominal temperature of the calibration scheme can be cancelled by using the heating and cooling results.
*Explanation #3*: If the calibration scheme is well designed to be more efficient, one set of calibration motions can be finished in a shorter period and the temperature change during the scheme will be smaller. Therefore the disturbance of the temperature change to the calibration result at the temperature point can be mitigated accordingly.

Both *Improveme*n*t #1* and *#2* are utilized to mitigate *Issue #1*, while *Improvement #2* and *#3* aim to ease *Issue #2*. With such improvements, the inherent problems of the Ramp method can be mitigated.

### 3.2. Calibration Scheme

**Figure 1.**
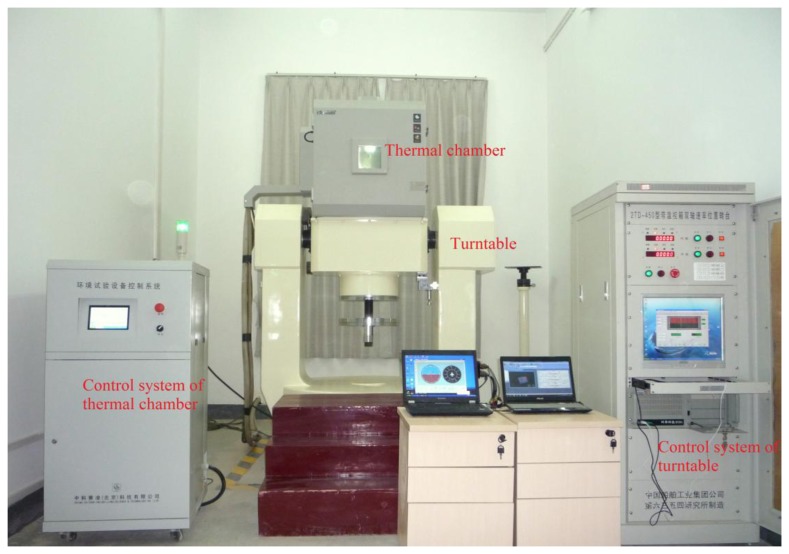
Thermal calibration equipment.

A dual-axis position turntable equipped with a thermal chamber (see Figure 1) located at Wuhan University is used to calibrate the IMU sensor errors over a wide temperature range. The performance characteristics of both the turntable and the thermal chamber are shown in Table 1.

The turntable is accurate enough to provide reference for the calibration of low-grade IMUs. Besides, there are high-precision locating pins on the mounting plate of the turntable to keep the IMUs axes align with the turntable axes.

**Figure 2.**
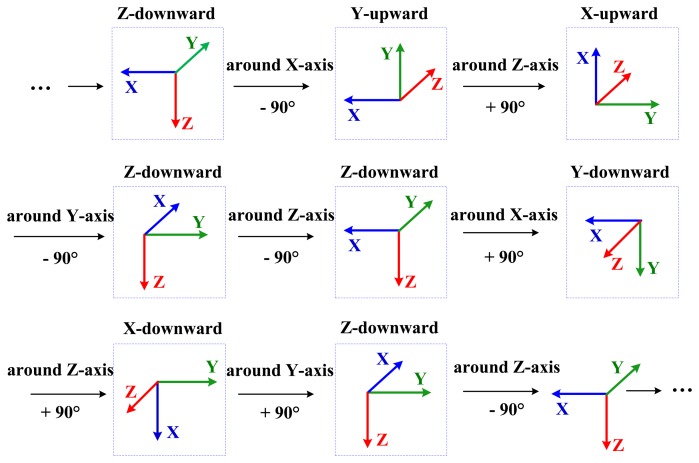
IMU actions in an 8-step calibration scheme.

Considering both the characteristics of the equipment and the calibration efficiency, the following 8-step calibration scheme is designed (see Figure 2). This scheme allows the IMUs to experience all necessary orientations and rotations with minimum calibration actions.

In each calibration scheme, each sensing axis of every sensor is pointed alternately up and down precisely (except z-axis due to the mechanical structure of the turntable), and the IMUs are rotated around each gyro axis both clockwise and counter-clockwise at accurately known angles. There are in total eight static positions and eight rotations.

In our test, each static position takes 10 s and the rate of rotation is about 10 deg/s, *i.e.*, about 9 s for each rotation. Due to such settings, the calibration scheme is highly efficient, and can provide enough calibration accuracy for low-grade IMUs, as shown in Section 3.3 for the calculation of the calibration errors caused by sensor noises. Each calibration scheme takes only about (10 + 9) × 8 = 152 s to estimate a full set of IMU sensor errors.

During the whole calibration process, the thermal chamber keeps on changing its temperature linearly. At the same time, the turntable repeats the calibration scheme in Figure 2 again and again. Due to the efficiency of the scheme, we obtain abundant data of IMU sensor errors together with temperatures over the whole temperature cycle.

### 3.3. Sensor Error Models and Calibration Computation

The error models for the accelerometers and the can be written as

(1) $$\hat{f}=b_{a}+S_{a}f+N_{a}f+v_{a}$$

(2) $$\hat{\omega }=b_{g}+S_{g}\omega +N_{g}\omega +v_{g}$$

where **f̂** and **ω̂** are the error vectors of the accelerometer-derived specific forces and the gyro-derived angular velocities, **f** and **ω** are the true specific forces and the true angular velocities, **b***_a_* and **b***_g_* are the biases of the accelerometers and the gyros, **S***_a_* and **S***_g_* are the diagonal matrices containing the scale factor errors, **N***_a_* and **N***_g_* are the skew-symmetric matrix containing the non-orthogonalities, ν*_a_* and ν*_g_* represent the a ccelerometer and gyro noises.

In each calibration scheme, the IMU sensor errors, including biases, scale factor errors and non-orthogonalities, are determined. The IMU data is sufficiently used: the static data (*i.e.*, the IMU outputs at eight static positions) is used to calibrate the gyro biases and all the accelerometer errors, while the dynamic data (*i.e.*, the IMU outputs during eight rotation steps) is used to calibrate gyro scale factors and non-orthogonalities. The accelerometer errors can be estimated by the least-square method [2], while the gyro errors are estimated through a two-step method [24]. The details of calibration computations are shown in Appendix A.

The calibration errors caused by sensor noises can be calculated as follows [25]. Since there are eight static positions, gyro noises can lead to calibration error of gyro biases $\sigma_{b_{g}-\text{noise}}=\frac{\text{ARW}}{\sqrt{8}.\sqrt{t_{\text{static}}}}(1\sigma )$, where *_ARW_* are the angular random walk of gyros, *t*_static_ is the time for each static position. Similarly, the calibration error of accelerometer errors caused by accelerometer noises can be calculated. For the calibration of gyro scale factors and non-orthogonalities, there are at least two rotations (positive and negative) for each gyro. Thus the gyro noise can cause calibration error of gyro scale factors or non-orthogonalities $\sigma_{s_{g}-\text{noise}}=\frac{\text{ARW}\cdot \sqrt{t_{\text{rotate}}}}{\sqrt{2}\cdot 90{}^{\circ}}$, where *t*_rotate_ is the time for each rotation. For example, assuming the gyro ARW is $0.05\deg/s/\sqrt{\text{HZ}}$ (refer to that of typical MEMS gyros), the errors caused by gyro noise are about 20 deg/h for gyro biases, and about 1,200 ppm for gyro scale factors or non-orthogonalities, which is much smaller than the thermal drifts of the sensor errors of low cost MEMS gyros.

### 3.4. Establishment of the Thermal Variation Models

Based on the calibrated IMU sensor errors at different temperatures, the curve fit method was used to determine the continuous global temperature compensation models (*i.e.*, the relationship between the sensor errors, *i.e.*, biases, scale factors and non-orthogonalities, and the temperature). A 3rd-order polynomial is used to fit the calibration results obtained from each scheme:

(3) $$W(T)=A_{0}+A_{1}\cdot T+A_{2}\cdot T^{2}+A_{3}\cdot T^{3}$$

where *W*(*T*)is the calculated value of the sensor errors, *T* is the IMU core temperature in real operation process, *A*_0_, *A*_1_, *A*_2_ and *A*_3_ are the fitting parameters. The established thermal model can be used to calculate the thermal drifts of the IMU sensor errors at each temperature and then remove such thermal drifts from the IMU outputs.

## 4. Thermal Calibration Tests and Results

### 4.1. Temperature Change Strategy

**Figure 3.**
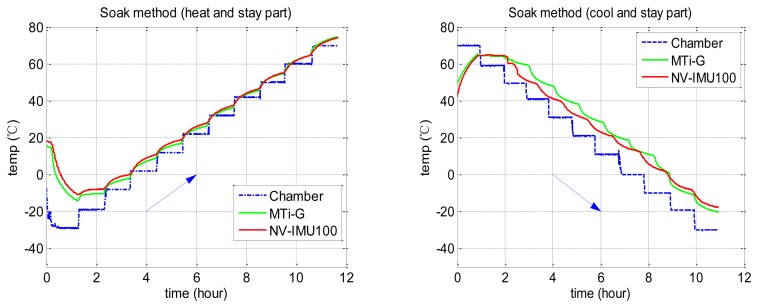
Temperature profile in Soak method test.

**Figure 4.**
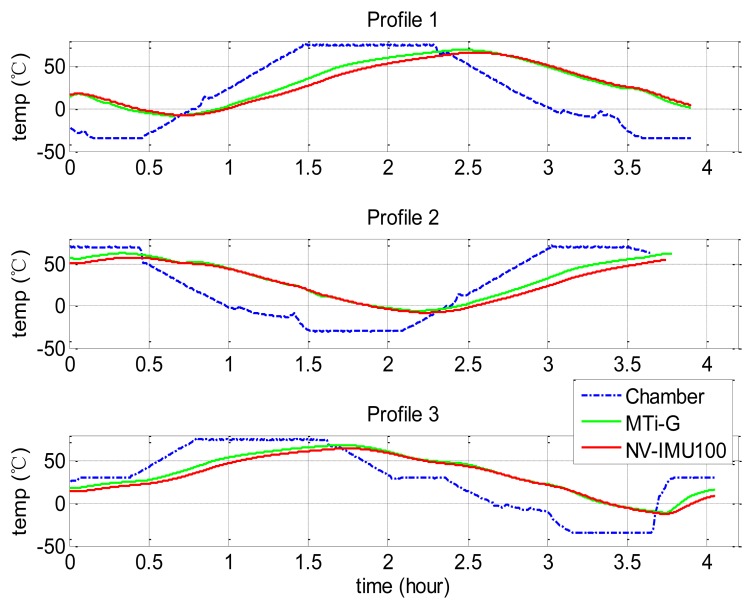
Temperature profiles in proposed method tests.

To evaluate the accuracy of the proposed method, we carried out the tests of the conventional Soak method to get the reference. The IMU calibration results at more than 10 temperature points were collected to provide accurate reference value, even though the Soak method does not need so many points and so much time for common uses. We also designed three different chamber temperature profiles to test the repeatability of the proposed method. Table 2 describes the detail of the chamber temperature changes. Figures 3 and 4 show the temperature profiles and how the internal temperatures of the two tested IMUs (Xsens MTi-G and NV-IMU100) changed. In both Figures 3 and 4, the dashed lines are the chamber temperatures, while the solid lines are the temperature provided by the internal temperature sensors of the two tested IMUs. It is clear to see the temperature lags of the IMU cores. Besides, Figure 3 shows that the IMU temperatures were higher than the chamber temperature after stabilization due to the heat generated inside the IMUs.

### 4.2. IMUs Used in the Tests

**Figure 5.**
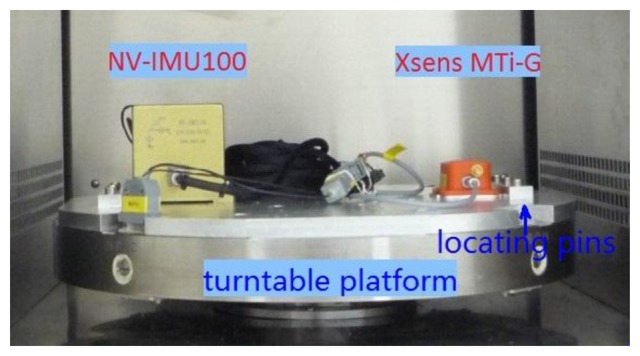
Two tested MEMS IMUs and the installation on the mounting plate of the turntable.

Two MEMS IMUs, *i.e.*, Xsens MTi-G [26] and NV-IMU100 [27], were chosen to be the representative of low-grade IMUs to be tested. Their photos in the test and their characteristics are illustrated in Figure 5 and Table 3, respectively. Here we used the raw sensor data of MTi-G (without factory compensation). The NV-IMU100 has three vibrating ring gyros, and its output data had been partly compensated after factory calibration.

### 4.3. Calibration Results of MTi-G

**Figure 6.**
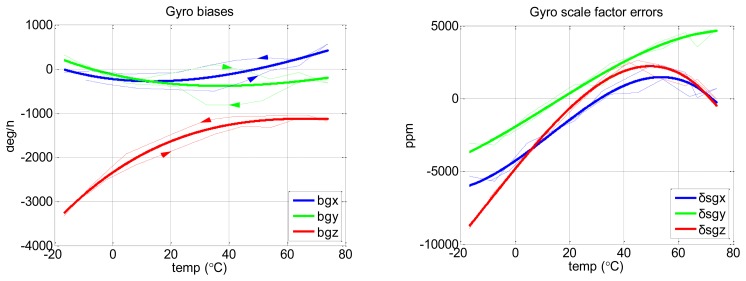
Thermal calibration results of MTi-G gyro biases and scale factor errors with Soak method. The thin solid lines are the results under separated heating and cooling processes (rightward and leftwards arrows indicate heating and cooling processes, respectively). The thick lines are the final curve fitted result (3rd-order polynomial fit).

Figure 6 shows the results of the biases and scale factor errors of gyros with the Soak method, as the example results of this conventional method.

The thin solid lines in Figure 6 show that even when using the Soak method, there were some non-repeatabilities between the results during Part 1 (from −30 to 70 °C with 10 °C step) and Part 2 (from 70 to −30 °C with 10 °C step;. After checking the temperature data of the IMUs, we found that it is because the sensor temperature inside the IMUs had not been fully stabilized even after the chamber temperature outside the IMUs had been stabilized for 1 h. This means that more than 1 h is needed for each temperature point to get the ideal result of the Soak calibration method for MTi-G. However, we cannot afford more than 1 h (e.g., 2 h; for each point for in-lab tests. Thus, we finally used 1 h per point, but fitted the results of Part 1 and Part 2 (*i.e.*, heating and cooling profiles) at the same temperature point to calculate the final IMU sensor errors at this temperature. This is the best result we can get and was regarded as the reference for the evaluation of the proposed method.

**Figure 7.**
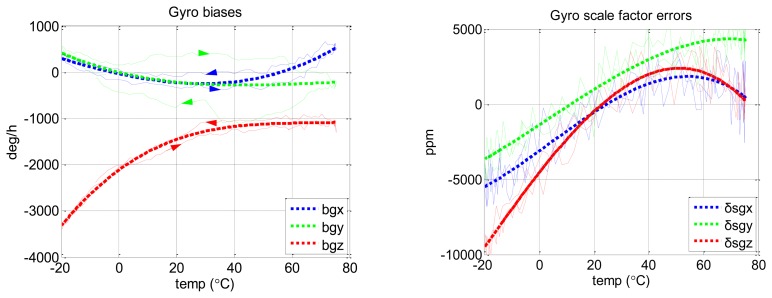
Thermal calibration results of MTi-G gyro biases and scale factor errors with proposed method. The thin dashed lines show the raw calibration results (*i.e.*, the result of each calibration scheme). Rightward and leftwards arrows indicate heating and cooling processes, respectively. The thick dashed lines are the final fitted results (3rd-order polynomial fit).

Figure 7 shows the results of gyro biases and scale factor errors with the proposed method, as the example results of this new method.

The raw results of the proposed method (the thin dashed lines) have some non-repeatabilities during heating and cooling processes. This might be caused by different temperature change rates. However, after fitting the raw results, we got the final result (the thick dashed line), which fit well with the reference result in Figure 6.

**Figure 8.**
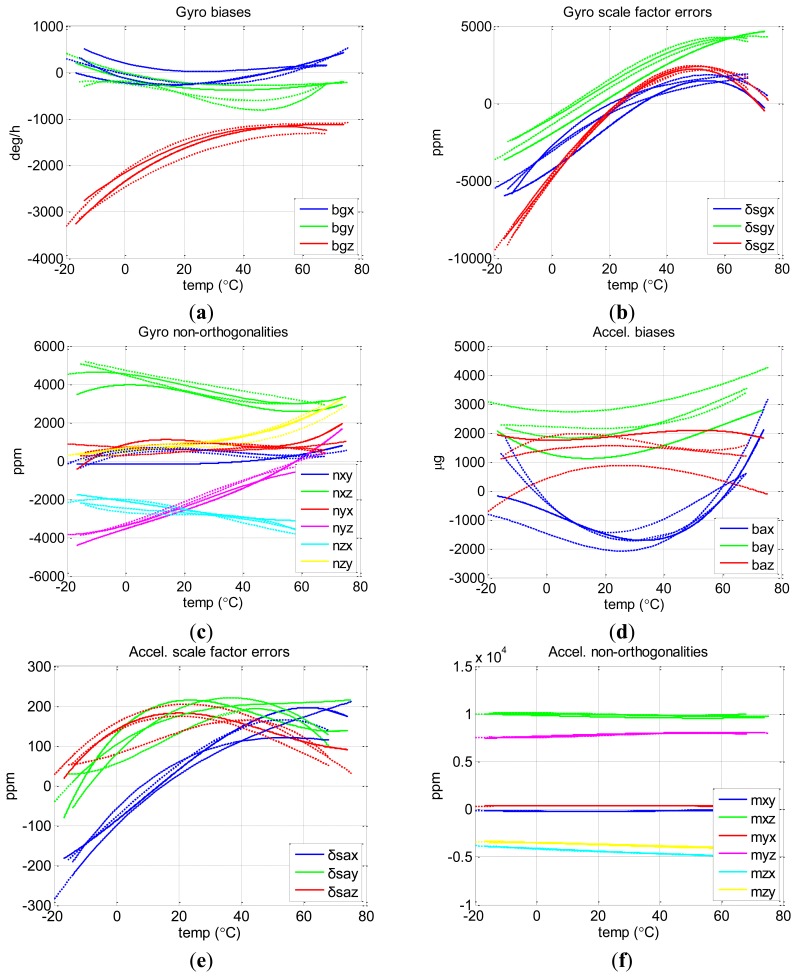
Thermal calibration results of MTi-G. In each subplot, three dashed lines correspond to the results of the proposed method with three different temperature profiles, and the solid line is the result of the Soak method.

To test the repeatability of the proposed method, we run the chamber temperature as the three different profiles described in Table 2. Figure 8 shows the final results of the three tests of the proposed method with different profiles (three dashed lines) as well as that of the Soak method (the solid line). Figure 8a–f show the curves of biases, scale factor errors, and non-orthogonalities of gyros and those of accelerometers.

It is clear that the sensor errors of MTi-G varied significantly with temperature, especially the gyro biases and scale factors. Over the entire temperature range, the changes reached 2,000 deg/h, 10,000 ppm for MTi-G gyro biases and scale factor errors, and 3,000 μg and 400 ppm for accelerometer biases and scale factor errors. There were also changes of about 5,000 ppm and 1,000 ppm for gyro and accelerometer non-orthogonalities, which were beyond our intuition. These curves indicate that thermal calibration is important not only for biases and scale factors, but also for non-orthogonalities, at least in the case of MEMS IMUs.

To evaluate how much the final results provided by the proposed method (the three dashed lines) fit with that of the Soak method (the solid line), we picked some typical temperature points and calculated the Root Mean Square (RMS) errors (using the results of the Soak method as reference) of the three temperature profiles, as shown in Table 4. The first 12 rows are the results of the gyro biases, scale factor errors and non-orthogonalities, while the last 12 rows are those of the accelerometers of MTi-G. Columns 2–6 show the RMS of the sensor errors under typical temperature points and column 7 picks up the max values among them.

Over the entire temperature range, their RMS with respect to the result of the Soak method reached 360 deg/h, 1,500 ppm and 1,300 ppm for gyro biases, scale factors and non-orthogonalities; and 1,200 μg, 100 ppm, and 400 ppm for accelerometer biases, scale factor errors and non-orthogonalities. The accuracy of the proposed method is shown in Table 5. Also, the original thermal drift levels of each sensor error over −10 ∼ +70 °C are shown to make a comparison.

The overall accuracy levels are much lower than the levels of the original thermal drifts of the MEMS sensor errors. The proposed calibration method has enough accuracy to significantly reduce the original temperature variations of the MEMS IMU sensor errors.

The thermal calibration results of MTi-G also indicate that not all the sensors errors can be reduced significantly through calibration, since the errors of each sensor are randomly distributed in the range of its specifications and some errors may be small originally. What we can expect is that through calibration and compensation, the overall levels of IMU sensor errors can be reduce to a much lower level.

### 4.4. Calibration Results of NV-IMU100

**Figure 9.**
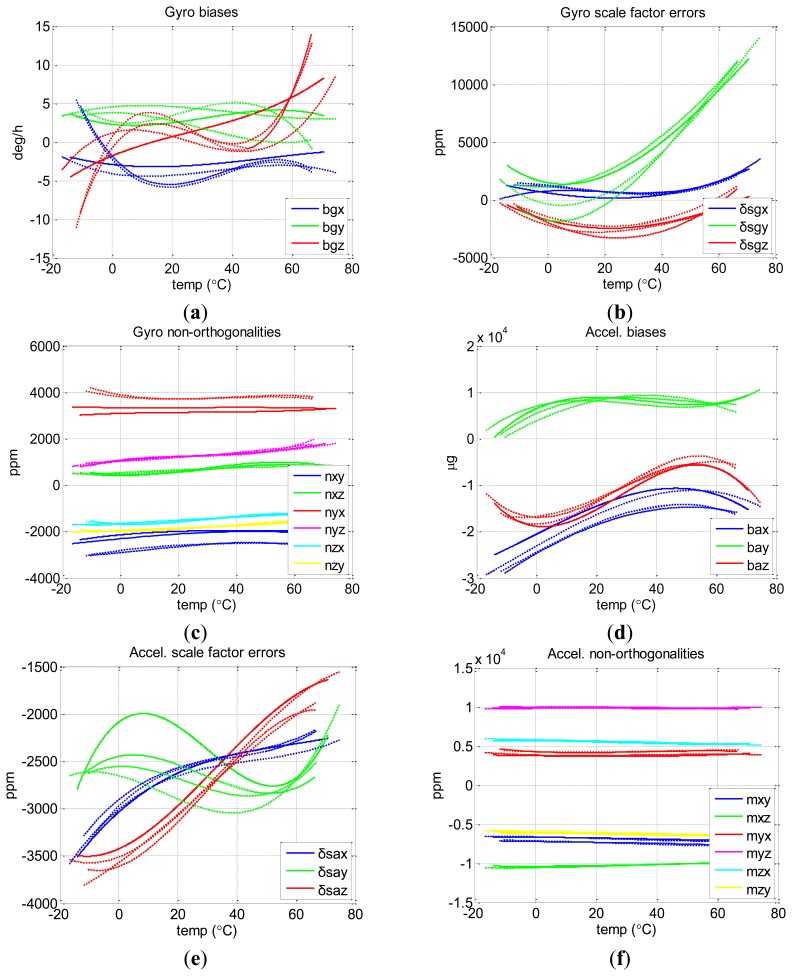
Thermal calibration results of NV-IMU100. In each subfigure, three dashed lines correspond to the results of the proposed method with three different temperature profiles, and the solid line is the result of the Soak method.

Similarly, we calibrated the thermal drift of NV-IMU100 with both the proposed method (using three different profiles) and the Soak method. Figure 9 shows the results provided by both the proposed method (the three dashed lines) and the Soak method (the solid line).

There were small gyro biases (less than 15 deg/h) due to the symmetry of vibrating ring gyros and the factory compensation. However, the change for gyro scale factor (e.g., y-axis) exceeded 10,000 ppm. These characteristics matched the inherent feature of the vibrating ring gyros.

Like Table 4, Table 6 shows the RMS errors (using the results of the Soak method as reference) of the proposed method at some typical temperature points.

Over the entire temperature range, the RMS errors reached 5 deg/h, 2,200 ppm and 900 ppm for gyro biases, scale factor errors and non-orthogonalities; and 4,200 μg, 700 ppm, and 600 ppm for accelerometer biases, scale factor errors and non-orthogonalities, respectively. The accuracy of the proposed method is shown in Table 7 as well as the original thermal drift ranges of each sensor error over −10 ∼ +70 °C. The overall accuracy levels are much lower than the levels of the possible thermal drift of the MEMS sensor errors.

The proposed thermal calibration method can provide results close to those of the conventional Soak method, and can significantly reduce the calibration time. Thus it is especially useful for MEMS IMUs, which require low cost and efficient calibration. Please note that the thermal calibration accuracy not only depend on the calibration method itself, but also is affected by the stochastic errors of the calibrated inertial sensors. This can explain why the same method got different calibration accuracies for the two tested IMUs. For example, the calibration accuracy of gyro biases of NV-IMU100 can reach 5 deg/h because the original gyro biases are relatively stable. To further investigate the effect of thermal calibration, another kind of tests called thermal compensation tests were performed, as described in Section 5.

## 5. Thermal Compensation Tests and Results

### 5.1. Test Description

The thermal compensation tests were designed to check how much the performance of the inertial sensors can be improved by compensating their outputs with the established thermal models. There were totally six tests, including three dynamic tests and three static tests, which are described in Table 8. For each test, the thermal chamber was utilized to provide different temperature environment (from −10 °C to +70 °C;, and the turntable was used to provide precisely known reference. Every epoch of IMU outputs was corrected using the full set of IMU sensor errors provided by the thermal model at the same temperature (*i.e.*, the thermal compensation). Then the effect of the thermal compensation was evaluated by comparing the output errors (*i.e.*, the difference between the sensor outputs and the reference input) before and after the thermal compensation. To evaluate the performance of all the sensors before and after compensation, every accelerometer axis was kept on pointing downwards precisely for about an hour respectively. Besides, the IMUs were kept on rotating around each gyro axis at an angular rate of 10 deg/s, which was set according to the dynamic of land vehicles, for about an hour. The whole process took about six hours. In each test, we focused on the output error of one sensor. For example, we investigated the x-axis gyro output with a reference input of 10 deg/s in the dynamic test 1; and investigated the x-axis accelerometer output with a reference input of the local gravity in the static test 1. The IMU outputs were averaged during each second to reduce the impact of the noises. The thermal models established through thermal calibration test 2 using the 2nd temperature profile were used in this test, since the results provided by test 2 seemed to have the largest differences comparing with those of the Soak method. The parameters of the 3rd order polynomial thermal models of applied in the compensation tests, *i.e.*, *A*_0_, *A*_1_, *A*_2_ and *A*_3_ in Equation (3), for the sensor errors of the two tested IMUs are shown in Table 9. The test results of MTi-G and NV-IMU100 are shown in Section 5.2 and 5.3, respectively.

### 5.2. Compensation Results of MTi-G

**Figure 10.**
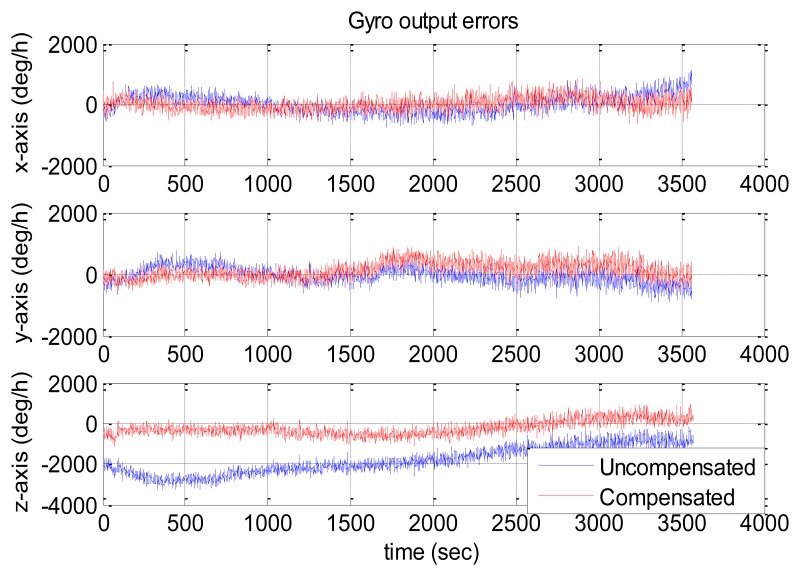
MTi-G gyro output errors with and without compensation.

**Figure 11.**
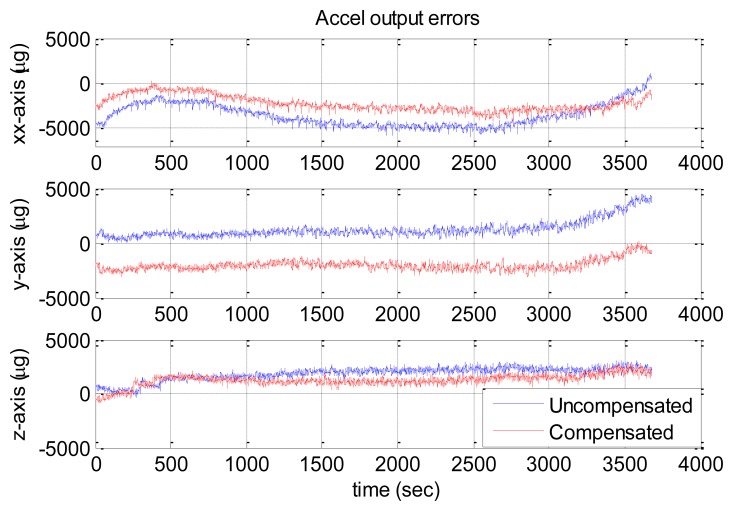
MTi-G accelerometer output errors with and without compensation.

Figures 10 and 11 show the compensation results of MTi-G. The blue and red lines are the output errors without and with the compensation using the established thermal models.

It is clear that the original output error of the z-axis gyro was significantly larger and changed with some long-term trends. After compensation, such error was significantly reduced, and the long-term trends were eliminated. However, there was no significant improvement for x-axis and y-axis gyros, since their original output errors were relatively small. As for the accelerometers, the improvement was significant along x-axis and z-axis. However, the y-axis accelerometer got an output error of about 2,000 μg after compensation. As has been explained in Section 4.3, not all the sensors errors can be reduced significantly through the thermal compensation, because the thermal drift level of each sensor are randomly distributed in the range of its specifications and some may be small originally. The thermal compensation can reduce the overall thermal drifts of the IMU outputs to a much lower level.

**Figure 12.**
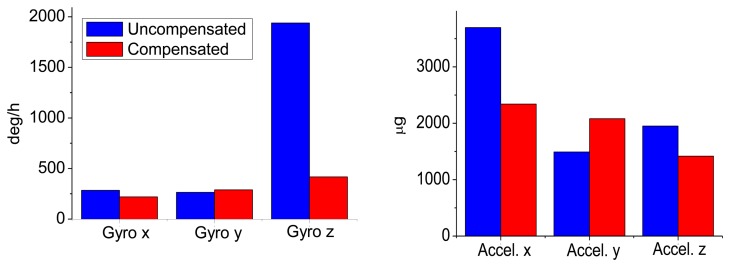
RMS values of MTi-G output errors with and without compensation.

The statistical values (*i.e.*, the mean and the RMS values) of the IMU output errors over the entire temperature range were calculated and shown in Table 10. Without compensation, the RMS values were 279, 197 and 2,167 deg/h for gyros and 3,697, 1,097 and 11,130 μg for accelerometers. After the compensation, the RMS values became 189, 254 and 418 deg/h for gyros and 2,342, 2,259 and 1,055 μg for accelerometers. Both the gyro and accelerometer along the z-axis and x-axis were improved significantly, while those along the y-axis became worse. Generally speaking, with the thermal compensation, the output errors of MTi-G were reduced to within 420 deg/h and 2,400 μg for gyros and accelerometers over the entire temperature range. To make it clear, the RMS values were shown in Figure 12. The blue and red bars were the values without and with thermal compensation, respectively.

### 5.3. Compensation Results of NV-IMU100

**Figure 13.**
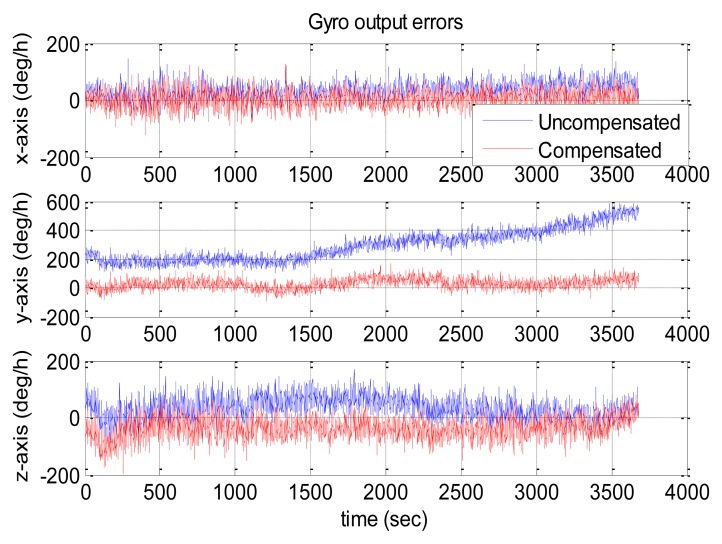
NV-IMU100 gyro output errors with and without compensation.

**Figure 14.**
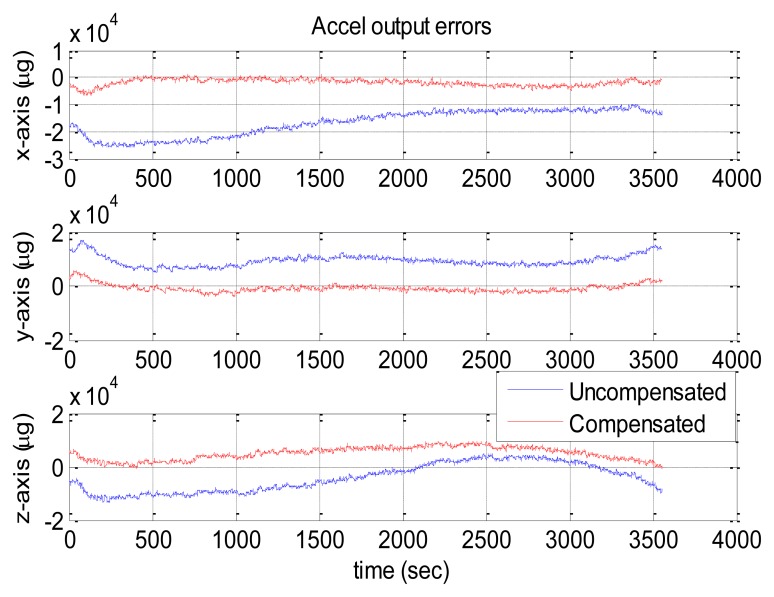
NV-IMU100 accelerometer output errors with and without compensation.

**Figure 15.**
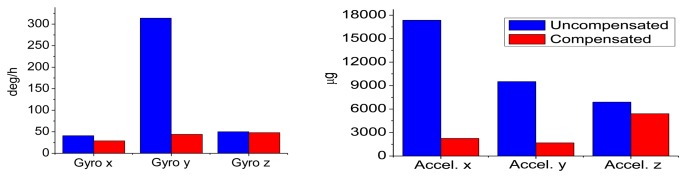
RMS values of NV-IMU100 output errors with and without compensation.

Similarly, Figures 13 and 14 show the NV-IMU100 output errors without and with thermal compensation. The NV-IMU100 has small gyro biases due to the symmetry of the vibrating ring gyros and the factory compensation. However, there were also gyro output errors caused by the scale factor errors and non-orthogonalities, especially for the y-axis gyro, whose scale factor varied for thousands of ppm over the entire temperature range. Consequently, the gyro output error reached several hundred deg/h when the input angular velocity was 10 deg/h. Clearly, such output errors were reduced significantly after the thermal compensation. The errors of accelerometers, especially the x-axis and y-axis accelerometers, were also reduced dramatically. The systematic errors in the outputs were corrected, and long-term trends in the outputs were eliminated. The statistical values of the NV-IMU100 output errors were shown in Table 11, and the RMS values were illustrated in Figure 15.

## 6. Conclusions

An efficient thermal calibration method is proposed to investigate the thermal drift of a full set of IMU sensor errors (*i.e.*, biases, scale factor errors and non-orthogonalities) over the entire temperature range in 4 h. Results of thermal calibration tests with two MEMS IMUs over the temperature range of −10 ∼ +70 °C showed that the proposed method provided results close to those of the conventional Soak method, and significantly reduced the calibration time. The accuracy of the proposed method over the entire temperature range (reference to the Soak method; was shown in Table 12.

Results of the separate thermal compensation tests showed that the performance of the inertial sensors can be significantly improved by compensating their outputs using the established thermal models. The max RMS values of the IMU output errors without and with thermal compensation were shown in Table 13.

Due to the high efficiency and sufficient accuracy of the proposed thermal calibration method, it is especially useful for both factory and lab calibrations of low-cost IMUs (e.g., MEMS), which require low-cost and efficient calibration. It can also promote better utilizations of low-cost MEMS-based sensors and IMUs.

## Figures and Tables

**Table 1.** Performance of thermal calibration equipment.

**Turntable**	
Principal axis rotation range	Continuous infinite
Principal axis angular position accuracy	±5″
Tilting axis rotation range	±95°
Tilting axis angular position accuracy	±5″
Non-orthogonalities between axes	±5″
**Thermal Chamber**	

Temperature range	−55 ∼ +100 °C
Temperature change rate	±0.1 ∼ ±5 °C/min linear

**Table 2.** Description of temperature changes in tests of both Soak and proposed method.

**Method**	**Chamber Temperature Changes**
Soak method (as reference: provides the best reference of IMU sensor errors we can get)	Consists of two parts: Part 1 (heat-and-stay part): increased from −30 °C to +70 °C using 10 °C steps (*i.e.*, step at −30, −20, (x02212)10, 0, 10, 20, 30, 40, 50, 60 and 70 °C). Kept fixed at each step for 1 h. Part 2 (cool-and- stay part;: decreased from +70 °C to (x02212;30 °C using 10 °C steps. Also 1 h per step.
Proposed method	Changed linearly at ±2 °C/min for a complete cycle over −35 °C ∼ +75 °C. Kept the temperature fixed for a period at both −35 °C and +75 °C to expand the temperature range inside the IMUs to nearly (x02212;10 °C ∼ +70 °C.
	A full calibration cycle took about hours.
	Used the following three profiles to test the repeatability of the proposed method.
Profile 1	25 °C → −35 °C (keep 30 min) → +75 °C (keep 60 min) →−35 °C (keep 30 min;
Profile 2	25 °C → +75 °C (keep 30 min) → −35 °C (keep 30 min)→+75 °C (keep 30 min;
Profile 3	25 °C →+75 °C (keep 40 min) → 25 °C (keep 20 min) →−35 °C (keep 30 min) → 25 °C (keep 30 min)

**Table 3.** Characteristics of tested IMUs.

**Characteristics**	**MTi-G**	**NV-IMU100**
Data Rate	100 Hz	166 Hz
Operating Range	−40∼85 °C	−40∼80 °C
Gyro Bias (1σ)	3,600 deg/h	4,000 deg/h
Gyro White Noise (ARW)	3.0 deg/√h	1.8 deg/√h
Gyro Scale factor error (1σ)	-	5,000
Accel. Bias (1σ)	2,000 μg	9,000 μg
Accel. White Noise (VRW)	0.002 m/s2/√Hz	0.01 m/s2/√Hz
Accel. Scale factor error (1σ)	3,000 ppm	2,000 ppm

**Table 4.** Errors (RMS) of MTi-G thermal calibration at typical temperature points (using the results of the Soak method as reference).

**Sensor Errors**	**−10 °C**	**10 °C**	**30 °C**	**50 °C**	**70 °C**	**Max**
bb_gx (deg/h)	353	214	137	87	158	353
b_gy (deg/h)	224	85	204	286	5	286
b_gz (deg/h)	215	182	144	114	130	215
δs_gx (ppm)	891	1,442	655	286	1,255	1,442
δs_gy (ppm)	906	1005	936	528	402	1,005
δs_gz (ppm)	256	307	155	180	953	953
n_xy (ppm)	364	778	612	157	361	778
n_xz (ppm)	1,070	393	429	525	259	1,070
n_yx (ppm)	353	512	265	39	949	949
n_yz (ppm)	345	271	375	209	1,224	1,224
n_zx (ppm)	412	307	83	339	1,071	1,071
n_zy (ppm)	145	96	108	221	287	287
b_ax (μg)	1,050	367	271	442	662	1,050
b_ay (μg)	801	1,176	1,166	1,049	949	1,176
b_az (μg)	1,146	639	688	1,030	1,166	1,166
δs_ax (ppm)	20	23	17	44	50	50
δs_ay (ppm)	21	64	26	33	45	64
δs_az (ppm)	18	39	19	21	34	39
m_xy (ppm)	16	21	17	25	33	33
m_xz (ppm)	142	229	250	259	310	310
m_yx (ppm)	37	28	32	36	34	37
m_yz (ppm)	29	104	66	31	121	121
m_zx (ppm)	12	49	21	20	91	91
m_zy (ppm)	85	41	55	67	123	123

**Table 5.** Calibration accuracy of proposed method (reference to Soak method) and the original thermal drift levels of the IMU errors over the whole temperature range (with MTi-G).

**Sensor Errors**	**Accuracy of Thermal Calibration**	**Original Drifts over−10 ∼ +70 °C**
Gyro biases	360 deg/h	2,100 deg/h
Gyro scale factor errors	1,500 ppm	11,000 ppm
Gyro non-orthogonalities	1,300 ppm	6,000 ppm
Accel. biases	1,200 μg	3,800 μg
Accel. scale factor errors	100 ppm	400 ppm
Accel. non-orthogonalities	400 ppm	1,000 ppm

**Table 6.** Errors (RMS) of NV-IMU100 thermal calibration at typical temperature points (using the results of the Soak method as reference).

**Sensor Errors**	**−10 °C**	**10 °C**	**30 °C**	**50 °C**	**70 °C**	**Max**
b_gx (deg/h)	5	1	1	0	2	5
b_gy (deg/h)	0	1	1	2	3	3
b_gz (deg/h)	3	2	1	4	4	4
δs_gx (ppm)	411	585	328	72	363	585
δs_gy (ppm)	2,143	2,039	1,310	372	190	2,143
δs_gz (ppm)	297	467	544	353	792	792
n_xy (ppm)	598	571	473	430	578	598
n_xz (ppm)	40	92	44	141	37	141
n_yx (ppm)	874	522	497	538	403	874
n_yz (ppm)	57	41	20	47	113	113
n_zx (ppm)	103	81	40	42	30	103
n_zy (ppm)	40	51	40	42	30	103
b_ax (μg)	4,133	3,520	3,417	3,036	1,337	4,133
b_ay (μg)	1,634	1,368	704	924	2,325	2,325
b_az (μg)	829	2,170	1,514	1,101	3,860	3,860
δs_ax (ppm)	95	62	49	68	90	95
δs_ay (ppm)	115	607	450	120	265	607
δs_az (ppm)	179	146	135	153	226	226
m_xy (ppm)	380	443	490	523	511	523
m_xz (ppm)	223	106	41	16	16	223
m_yx (ppm)	547	398	472	521	313	521
m_yz (ppm)	169	149	124	50	134	169
m_zx (ppm)	161	83	102	87	135	161
m_zy (ppm)	205	112	100	110	69	205

**Table 7.** Calibration accuracy of proposed method (reference to Soak method) and the original thermal drift ranges of the sensor errors over the whole temperature range (with NV-IMU100).

**Sensor Errors**	**Accuracy of Thermal Calibration**	**Original Drifts over −10 ∼ +70 °C**
Gyro biases	5 deg/h *	13 deg/h
Gyro scale factor errors	2,200 ppm	10,000 ppm
Gyro non-orthogonalities	900 ppm	1,000 ppm
Accel. biases	4,200 μg	14,000 μg
Accel. scale factor errors	700 ppm	1,800 ppm
Accel. non-orthogonalities	600 ppm	600 ppm

* The NV-IMU100 has three vibrating ring gyros, and the thermal drifts of its gyro biases had been partly compensated by factory calibration.

**Table 8.** Descriptions of the six thermal compensation tests.

**Motions of the IMUs (Provided by the Turntable)**	
Dynamic test 1	IMUs kept rotating around their x-axis at 10 deg/s.
Dynamic test 2	IMUs kept rotating around their y-axis at 10 deg/s.
Dynamic test 3	IMUs kept rotating around their z-axis at 10 deg/s.
Static test 1	IMUs kept static with x-axis pointed upwards.
Static test 2	IMUs kept static with y-axis pointed upwards.
Static test 3	IMUs kept static with z-axis pointed upwards.
**Chamber Temperature**	

Before each test, the temperature was stabilized at −10 °C;	
During each test, the temperature was changed from −10 °C to 70 °C in one hour.	

**Table 9.** The parameters of the 3rd order polynomial thermal models of the IMU errors applied in the thermal compensation tests, *i.e.*, *A*_0_, *A*_1_, *A*_2_ and *A*_3_ in Equation (3). The thermal models can be used to calculate the thermal drifts of the IMU sensor errors at each temperature and then remove such thermal drifts from the IMU outputs.

**Sensor Errors**	**MTi-G**				**NV-IMU1**			

	***A*_0_**	***A*_1_**	***A*_2_**	***A*_3_**	***A*_0_**	***A*_1_**	***A*_2_**	***A*_3_**
b_gx (deg/h)	−31	−13.65	0.177	0.00142	−4	−0.06	0.003	−3.24712
b_gy (deg/h)	1	−15.33	0.260	−0.00136	5	0.03	−0.002	1.56754
b_gz (deg/h)	−2,112	45.24	−0.682	0.00352	1	0.10	−0.009	0.00012
δs_gx (ppm)	−3,108	1.31	0.232	−0.01783	752	14.18	−1.288	0.02144
δs_gy (ppm)	−1,380	119.62	0.107	−0.00931	−377	−44.18	4.715	−0.02033
δs_gz (ppm)	−4,541	232.23	−1.134	−0.01477	−2,211	71.96	2.405	−0.01435
n_xy (ppm)	611	17.20	−0.906	0.00896	−2,304	11.76	−0.069	−0.00083
n_xz (ppm)	4,527	−18.12	−0.733	0.01023	483	3.40	0.234	−0.00288
n_yx (ppm)	701	−8.14	0.077	0.00111	3,344	−0.77	0.053	−0.00069
n_yz (ppm)	−3,371	39.47	0.620	−0.00810	1,081	11.43	−0.229	0.00277
n_zx (ppm)	−2,017	−10.81	−0.783	0.00761	−1,699	3.48	0.201	−0.0023
n_zy (ppm)	593	6.73	−0.293	0.00813	−1,939	4.98	−0.008	0.00038
b_ax (μg)	−1,472	−37.02	0.120	0.01575	−23,074	365.76	−0.808	−0.03441
b_ay (μg)	2,758	−6.76	0.443	−0.00128	7,069	169.46	−7.247	0.07519
b_az (μg)	411	38.25	−0.876	0.00360	−16,897	−27.51	13.616	−0.17003
δs_ax (ppm)	−100	7.71	−0.073	0.00034	−3,003	25.52	−0.472	0.00350
δs_ay (ppm)	102	5.25	−0.090	0.00055	−2,650	−8.41	−0.361	0.00814
δs_az (ppm)	157	4.32	−0.108	0.00038	−3,509	12.56	0.565	−0.00513
m_xy (ppm)	−174	−1.77	0.026	0.00052	−6,614	−6.55	−0.221	0.00287
m_xz (ppm)	9,996	−1.75	−0.129	0.00149	−10,539	4.26	0.195	−0.00179
m_yx (ppm)	372	1.50	−0.037	−0.00003	3,858	−10.07	0.429	−0.00387
m_yz (ppm)	7,648	8.42	0.017	−0.00088	9,956	5.03	−0.257	0.00261
m_zx (ppm)	−4,158	−15.85	0.095	−0.00034	5,713	−11.62	0.127	−0.00103
m_zy (ppm)	−3530	−7.14	−0.032	0.00018	−5912	−4.28	−0.109	0.00071

**Table 10.** Statistical results of MTi-G output errors with and without compensation.

**Sensors**	**Compensated**		**Uncompensated**	
	
	**RMS**	**Mean**	**RMS**	**Mean**
Gyro x (deg/h)	220	15	284	23
Gyro y (deg/h)	289	124	264	−37
Gyro z (deg/h)	418	−279	2164	−1974
Accel. x (μg)	2341	−2147	3697	−3287
Accel. y (μg)	2081	−2023	1490	1256
Accel. z (μg)	1416	1219	1951	1852

**Table 11.** Statistical results of NV-IMU100 output errors with and without compensation.

**Sensors**	**Compensated**		**Uncompensated**	

	**RMS**	**Mean**	**RMS**	**Mean**
Gyro x (deg/h)	29	4	41	29
Gyro y (deg/h)	44	27	314	294
Gyro z (deg/h)	48	−36	50	32
Accel. x (μg)	2253	−6105	17356	−16665
Accel. y (μg)	1687	−823	9516	9287
Accel. z (μg)	5410	4781	6894	−4403

**Table 12.** Accuracy of proposed method (reference to Soak method).

**Sensor Errors**	**Xsens MTi-G**	**NV-IMU100**
Gyro biases	360 deg/h	5 deg/h *
Gyro scale factor errors	1,500 ppm	2,200 ppm
Gyro non-orthogonalities	1,300 ppm	900 ppm
Accel. biases	1,200 μg	4,200 μg
Accel. scale factor errors	100 ppm	700 ppm
Accel. non-orthogonalities	400 ppm	600 ppm

* The NV-IMU100 has three vibrating ring gyros with stable bias inherently, and the thermal drifts of its gyro biases had been partly compensated by factory calibration.

**Table 13.** RMS of IMU output errors with and without thermal compensation.

**Sensors**	**Xsens MTi-G**		**NV-IMU100**	

	**Compensated**	**Uncompensated**	**Compensated**	**Uncompensated**
Max Gyro output error (deg/h)	418	2,164	48	314
Max Accel. output error (μg)	2,342	3,697	5,410	17,356

## Appendix A. Calibration computation of the proposed calibration scheme

### Estimation of Accelerometer Errors

To estimate a full set of accelerometer errors, the output of a triad of accelerometers is represented in matrix form:

(a.1) $$\begin{array}{ccc} \left[\begin{array}{c} {\tilde{f}}_{x}\\ {\tilde{f}}_{y}\\ {\tilde{f}}_{z} \end{array}\right]= & \underset{M}{\underset{︸}{\left[\begin{array}{cccc} s_{x} & m_{yx} & m_{zx} & b_{ax}\\ m_{xy} & s_{y} & m_{zy} & b_{ay}\\ m_{xz} & m_{yz} & s_{z} & b_{az} \end{array}\right]}} & \left[\begin{array}{c} f_{x}\\ f_{y}\\ f_{z}\\ 1 \end{array}\right] \end{array}$$

The diagonal *s* elements are the scale factors, the off diagonal *m* elements represent the non-orthogonalities and the *b* components are the biases. Assume S1, S2, S3, S4, and S5 are the IMU positions with x-axis upwards, x-axis downwards, y-axis upwards, y-axis downwards and z-axis downwards. Since the IMU is perfectly aligned with the turntable axes through the location pins on the mounting plate, the given specific forces of the five positions can be represented as follows:

(a.2) $$\begin{array}{ccccc} f_{1}^{\prime }=\left[\begin{array}{c} g\\ 0\\ 0 \end{array}\right], & f_{2}^{\prime }=\left[\begin{array}{c} -g\\ 0\\ 0 \end{array}\right], & f_{3}^{\prime }=\left[\begin{array}{c} 0\\ g\\ 0 \end{array}\right], & f_{4}^{\prime }=\left[\begin{array}{c} 0\\ -g\\ 0 \end{array}\right], & f_{5}^{\prime }=\left[\begin{array}{c} 0\\ 0\\ -g \end{array}\right] \end{array}$$

Then the design matrix can be denoted by **A** and the measured specific force of the accelerometer is denoted by **U**:

(a.3) $$A=\left[\begin{array}{c} f_{1}^{\prime }\\ 1 \end{array}\;\begin{array}{c} f_{2}^{\prime }\\ 1 \end{array}\;\begin{array}{c} f_{3}^{\prime }\\ 1 \end{array}\;\begin{array}{c} f_{4}^{\prime }\\ 1 \end{array}\;\begin{array}{c} f_{5}^{\prime }\\ 1 \end{array}\right]$$

(a.4) $$U=\left[\begin{array}{ccccc} u_{1} & u_{2} & u_{3} & u_{4} & u_{5} \end{array}\right]$$

In this case the column vector of the **U** matrix should be:

(a.5) $$\begin{array}{cc} u_{1}={\left[\begin{array}{c} {\tilde{f}}_{x}\\ {\tilde{f}}_{y}\\ {\tilde{f}}_{z} \end{array}\right]}_{x-\text{axis pointing up}}, & u_{2}={\left[\begin{array}{c} {\tilde{f}}_{x}\\ {\tilde{f}}_{y}\\ {\tilde{f}}_{z} \end{array}\right]}_{X-\text{axis pointing down}} \end{array}$$

**u_3_**, **u_4_** and **u_5_** are similar with **u_1_** and **u_2_** Then the **M** matrix can be estimated by the least-square method:

(a.6) $$M=U\cdot A^{T}{\left(A\cdot A^{T}\right)}^{-1}$$

#### Estimation of Gyro Biases

The gyro biases are calculated using static gyro outputs.

Let **ω̃***_i_* =[*ω̃_ix_ω̃_iy_ω̃_iz_*]*^T^i*=1,2,3,4,5 are the mean of gyro output angular velocities under static positions S1, S2, S3, S4, and S5. The elements of the vector represent the components along three axes. By averaging a couple of measurements, e.g., **ω̃**_1_ and **ω̃**_2_ or **ω̃**_3_ and **ω̃**_4_, the impact of the rotational angular velocity of the earth can be cancelled. The gyro biases are calculated as:

(a.7) $$b_{gx}=\frac{1}{4}\sum_{k=1}^{4}{\tilde{\omega }}_{kx},b_{gy}=\frac{1}{4}\sum_{k=1}^{4}{\tilde{\omega }}_{ky}$$

When the z-axis of the IMU is pointed downward, the z-axis gyro bias can be calculated using the gyro output minus the reference input when the gyro points vertically (which is not depend on the north finding of the turntable):

(a.8) $$b_{gz}={\tilde{\omega }}_{5x}+\omega_{e}\sin\varphi$$

where *ω_e_* is the rotational angular velocity of the earth, *φ* is the latitude.

#### Estimation of Gyro Scale Factors and Non-orthogonalities

Let **L̃***_i_* = [*L̃_ix_L̃_iy_L̃_iz_*]*^T^i*=1,2,3,4,5,6 be the integration of the gyro outputs when the IMU is rotated counterclockwise and clockwise around x-axis, counterclockwise and clockwise around y-axis, and counterclockwise and clockwise around z-axis for the angle of *L_ref_*, respectively. There are two factors that eliminate the impact of the rotational angular velocity of the earth: (a) In the designed calibration scheme, when the IMU is rotated around one axis both counterclockwise and clockwise, this axis is pointed toward its specific direction (as shown in Figure 2). (b) The rotation data (counterclockwise and clockwise) with the same time length is used for the calibration computation.

The gyro scale factors can be calculated as:

(a.9) $$\begin{array}{ccc} s_{gx}=\frac{{\tilde{L}}_{1x}-{\tilde{L}}_{2x}}{2L_{\text{ref}}}, & s_{gy}=\frac{{\tilde{L}}_{3y}-{\tilde{L}}_{4y}}{2L_{\text{ref}}}, & s_{gz}=\frac{{\tilde{L}}_{5z}-{\tilde{L}}_{6z}}{2L_{\text{ref}}} \end{array}$$

The value of *L_ref_* is 90 deg in our calibration scheme. The non-orthogonalities cause each axis to be affected by the signal of the other two axes. When the IMU is rotated around one axis, the gyro output of another axis will be affected by this rotation due to the non-orthogonalities between these two axes. The non-orthogonalities can be calculated as:

(a.10) $$\begin{array}{cccccc} n_{xy}=\frac{{\tilde{L}}_{1y}-{\tilde{L}}_{2y}}{2L_{\text{ref}}}, & n_{xz}=\frac{{\tilde{L}}_{1z}-{\tilde{L}}_{2z}}{2L_{\text{ref}}}, & n_{yx}=\frac{{\tilde{L}}_{3x}-{\tilde{L}}_{4x}}{2L_{\text{ref}}}, & n_{yz}=\frac{{\tilde{L}}_{3z}-{\tilde{L}}_{4z}}{2L_{\text{ref}}}, & n_{zx}=\frac{{\tilde{L}}_{5x}-{\tilde{L}}_{6x}}{2L_{\text{ref}}}, & n_{zy}=\frac{{\tilde{L}}_{5y}-{\tilde{L}}_{6y}}{2L_{\text{ref}}}, \end{array}$$

where *n_ij_* is the non-orthogonalities of i-axis to j-axis.
